# Self-Focusing SIMS Enables Quantitative Dopant Analysis in Nanoscale Silicon Devices beyond Conventional Spatial Resolution Limits

**DOI:** 10.1021/acsmeasuresciau.6c00086

**Published:** 2026-06-08

**Authors:** Valentina Spampinato, Alexis Franquet, Paul van der Heide

**Affiliations:** † Department of Chemical Sciences and Center for Colloid and Surface Science (CSGI), University of Catania, Viale A. Doria 6, 95125 Catania, Italy; ‡ 71351 IMEC, Kapeldreef 75, B-3001 Leuven, Belgium

**Keywords:** Self-Focusing SIMS, Nanoscale devices, Dopants, Quantification, Confined volumes

## Abstract

The continuous downscaling of semiconductor devices has created a pressing need for analytical methodologies capable of enabling process control in confined volumes and sub-100 nm features. Conventional secondary ion mass spectrometry (SIMS), while highly sensitive, lacks the spatial resolution required for such applications. Nevertheless, SIMS remains a powerful tool for the analysis of small features through the self-focusing (SF) SIMS concept, which exploits the formation of cluster ions that inherently localize chemical information within the region of interest. In this study, SF-SIMS is applied to the quantification of boron in patterned samples composed of boron-doped silicon fins with widths ranging from 500 to 20 nm, embedded in boron-doped silicon oxide. By selecting cluster ions that originate exclusively from the silicon fin region, the spatial resolution limitations of conventional SIMS are overcome without compromising the sensitivity. Using this approach, a boron implant with a peak concentration of ∼9 × 10^20^ at/cm^3^ was measured in the widest fins (500 nm), in good agreement with SRIM simulations and conventional SIMS methods, while a concentration of ∼4.5 × 10^20^ at/cm^3^ was obtained for the narrowest fins (20 nm). This study establishes SF-SIMS as a reliable, rapid, and preparation-free approach for dopant quantification in nanoscale semiconductor devices down to 20 nm.

## Graphical abstract

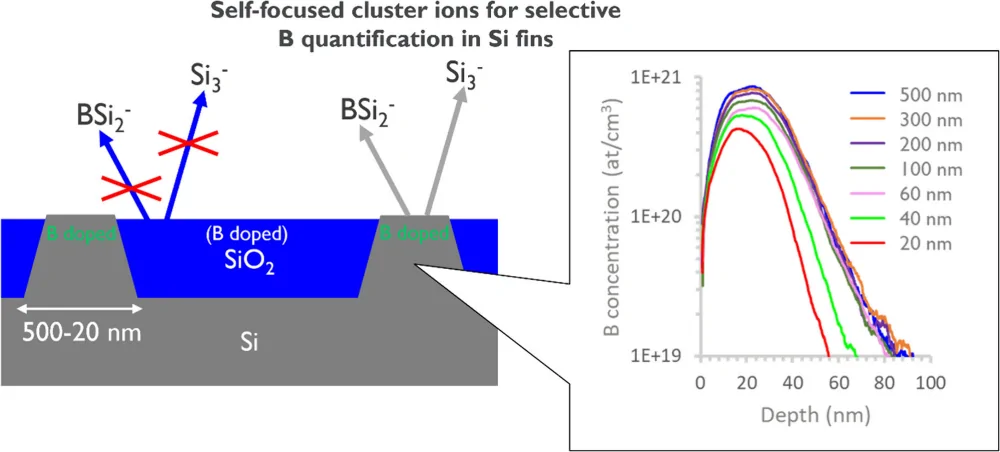

## 1 Introduction

As semiconductor technology continues to push the limits of miniaturization, device architectures have evolved far beyond simple planar designs. To sustain improvements in speed, power efficiency, and integration density, the industry is adopting complex structures such as multilayer stacks in complementary field-effect transistors,^1^ three-dimensional (3D) channel architectures,^2^ and confined geometries like nanosheets and forksheets^3–5^ to enable gate-all-around devices.^6,7^

Direct analysis of such intricate systems remains highly challenging; therefore, equivalent blanket samples are often used as proxies to infer the composition, defect characteristics, and dopant concentrations in the actual structures.

However, numerous studies have shown that the behavior of defects and dopants can differ significantly between blanket films and confined device geometries.^8^ This discrepancy arises mainly from the influence of the surrounding material (e.g., silicon oxide) and from differences in the surface-to-volume ratio, both of which strongly affect processes such as dopant diffusion.^9^ Consequently, blanket-based experiments no longer provide an accurate representation of nanoscale device behavior.

To support process development, advanced metrology approaches are increasingly required to enable the direct analysis of real device structures. Given the nanometer-scale dimensions of these architectures, characterization techniques must not only deliver chemical information but also achieve sufficient lateral and depth resolutions. Moreover, in the case of dopants, extremely high sensitivity is essential since the absolute number of dopant atoms per device feature is intrinsically limited.

Secondary ion mass spectrometry (SIMS) is among the most powerful techniques for probing dopant incorporation and studying diffusion, owing to its ability to provide quantitative measurements through the use of external standards, combined with high depth resolution and excellent sensitivity. However, the achievable lateral resolution, typically on the order of a few hundred nanometers for conventional dynamic SIMS, limits its applicability to relatively large structures, where the primary ion beam can be directly focused on the region of interest. In such cases, the sensitivity is often significantly reduced because of the limited volume analyzed.

Recent advances in SIMS instrumentation, including focused ion beam and high-resolution TOF-SIMS platforms developed by companies such as Raith and IONTOF, have extended the achievable lateral resolution to the sub-50 nm regime, enabling nanoscale chemical imaging and analysis. Nevertheless, these approaches generally involve a trade-off among lateral resolution, sensitivity, acquisition time, and statistical representativeness since the analysis is typically restricted to a limited number of devices or to very small sampled volumes.

Recent literature has demonstrated that the conventional SIMS lateral resolution can also be improved through the self-focusing SIMS (SF-SIMS) concept.^10–13^ With SF-SIMS, the information on relevance is attained by probing an ensemble of similar devices with a large beam, therefore improving the sensitivity, through the selection of characteristic cluster ions. These cluster ions are predicted to arise from nearest neighbor atoms (i.e., atoms within a 0.5 nm distance),^14^ and the compositional information measured is thus “self-focused” to the areas where all the constituents are simultaneously present. The SF-SIMS concept not only overcomes the SIMS limitations in terms of lateral resolution but also offers improved sensitivity thanks to the simultaneous measurement of many devices. Moreover, the method requires no prior sample preparation and is not tied to a specific SIMS setup, as consistent results have been demonstrated using various SIMS instruments.^10^

In this paper, the SF-SIMS approach is applied to quantify the boron doping level in silicon fins, with fin widths ranging from 500 to 20 nm, surrounded by boron-doped SiO_2_. Careful study of B-doped Si and B-doped SiO_2_ standards was initially carried out in order to identify the most suitable combination of cluster ions (one for the dopant and one for the matrix, respectively) to be used for the quantification. The most appropriate cluster ions were found to be BSi_2_^–^ for the dopant and Si_3_^–^ for the matrix since their intensity was negligible in the boron-doped silicon oxide reference.

Comparison with SRIM simulations and more conventional SIMS approaches, such as 1.5D SIMS^15^ and high lateral resolution SIMS,^16,17^ on the widest fins (i.e., 500 nm) confirmed the validity of the SF-SIMS approach. Interestingly, the B concentration was found to be lower for smaller fin dimensions.

Advantages and disadvantages of the above-mentioned approaches used (SF-SIMS, 1.5D SIMS, and high lateral resolution SIMS) are discussed, leading to the conclusion that SF-SIMS is a valid and unique approach to quantify dopants in very small devices (down to 20 nm) with high sensitivity and without requiring tedious and time-consuming sample preparation prior to the analysis.

## 2 Materials and Methods

### 2.1 Sample Description

The sample investigated in this study is composed by an array of silicon fins with variable width (from ∼20 to ∼500 nm, as shown in Figure 1a–d) surrounded by silicon oxide. The fins pattern was created by etching away portions of a bulk silicon wafer so that ∼300 nm tall fins with the desired widths and lengths (in this case ∼10 μm) would be generated. The areas where the silicon was etched away were filled with shallow trench isolation (STI) oxide to create the final structure, as depicted in Figure 1e in a transmission electron microscopy (TEM) cross section for the 500 nm wide fins. After the STI filling, B was implanted (3 × 10^15^ at/cm^2^, 5 kV, 7° tilt, 4-sided implant at room temperature), followed by a 450 °C oven anneal for 15 s in one case, and an extra 1150 °C millisecond laser anneal with 7 scans^18^ in the other case. In this study, we refer to the sample that underwent only the oven anneal as the “non-annealed” sample and the one that underwent the laser anneal as the “annealed” sample.

**Figure 1:**
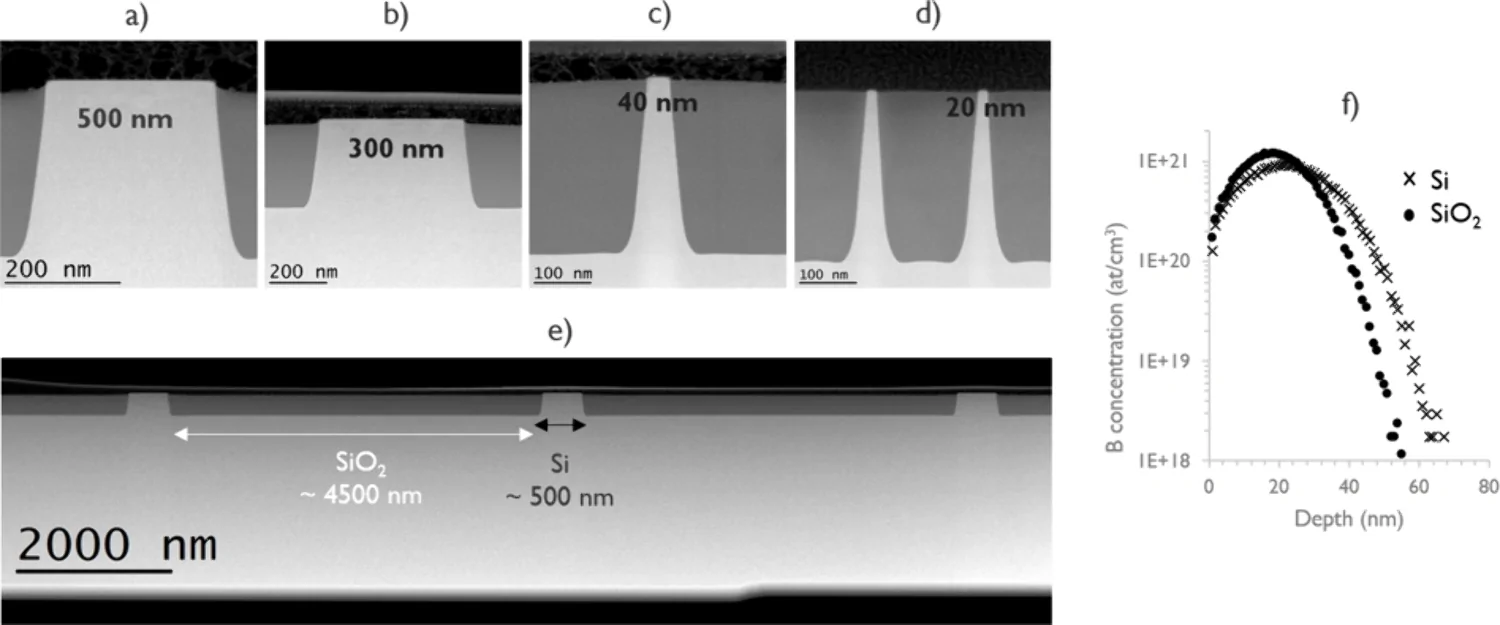
Cross-sectional TEM images for 500 (a, e), 300 (b), 40 (c), and 20 nm (d) wide fins, together with SRIM simulations for boron implant in silicon and silicon oxide (f).

### 2.2 SRIM

The boron implant was simulated using the stopping and range of ions in matter (SRIM)^19–22^ for both silicon^23^ (density ∼ 2.33 g/cm^3^) and silicon dioxide^24^ (density ∼ 2.65 g/cm^3^). For silicon, SRIM simulation returned an implant depth of ∼70 nm with a peak concentration of 9.3 × 10^20^ at/cm^3^ (taking into account the backscattered portion), corresponding to a dose of 2.93 × 10^15^ at/cm^2^, while for silicon oxide the implant depth is ∼60 nm with a peak concentration of 1.19× 10^21^ at/cm^3^, as shown in Figure 1f.

### 2.3 SIMS Analysis

The boron quantification on the STI-chemically etched sample was performed by means of an Atomika 4500 quadrupole instrument. As the primary source, O_2_^+^ at 500 eV was used (0° impact angle) and rastered over 300 × 300 μm^2^. No charge compensation was applied to the etched sample. We refer to this approach as “1.5D SIMS”, as stated in the Introduction.

For the high lateral resolution and SF-SIMS approaches, a ToF-SIMS NCS tool from IONTOF GmbH (Münster, Germany) with a 45° fixed configuration was used.

For the high lateral resolution measurements in positive polarity acquisition mode, the analysis was performed using a bismuth liquid metal cluster ion gun (LMIG) of 30 keV, using Bi_1_^+^ species in Fast Imaging (FI) mode (beam spot size of <150 nm) with a current of ∼0.2 pA on square areas of 15 × 15 μm^2^ with a resolution of 256 × 256 pixels. As the sputter species, an O_2_^+^ source at 500 eV (∼20 nA) was used and rastered over 200 × 200 μm^2^. A flood gun with 20 eV electrons was used for charge compensation, while O_2_ flooding (2.5 × 10^–6^ mbar) was used during the measurements in order to improve the ionic yield. For the high lateral resolution experiments conducted in negative polarity acquisition mode, while LMIG settings were kept identical to the ones used in positive polarity detection mode, Cs^+^ 350 eV (∼30 nA, 250 × 250 μm^2^) was utilized as the sputter beam, and no flood gun or flooding was applied. For the SF-SIMS approach, a Bi_1_^+^ LMIG at 15 keV (current ∼1 pA) was used in spectrometry mode (beam spot size ∼3 μm), rastered over 100 × 100 μm^2^. As the sputter species, a Cs^+^ source at 350 eV was used (∼30 nA), rastered over 250 × 250 μm^2^ areas.

Depth calibration of the SIMS depth profiles was attained by measuring the crater depth after sputtering, either with a profilometer (for the experiments carried out with the quadrupole SIMS) or with an in situ AFM (for the experiments carried out with the ToF-SIMS instrument).

Boron quantification in the silicon fins was performed using a relative sensitivity factor (RSF) extracted from an external blanket boron-doped silicon reference. For both the 1.5D SIMS and the high lateral resolution measurements acquired in positive polarity mode, B^+^ and ^30^Si^+^ ions were used as markers for the dopant and the matrix, respectively. In contrast, SF-SIMS quantification performed in negative polarity mode employed BSi_2_^–^ and Si_3_^–^ cluster ions as markers for the dopant and the matrix, respectively.

Boron quantification in the silicon oxide was performed by using an RSF extracted from an external blanket boron-doped silicon oxide reference. Quantification in the oxide was carried out only using the high lateral resolution approach, again employing B^+^ and ^30^Si^+^ ions as markers for the dopant and the matrix, respectively.

## 3 Results and Discussion

SIMS is a well-established technique for the quantification of dopants in microelectronic devices, thanks to the possibility to detect secondary ions specific for the dopant and secondary ions related exclusively to the matrix. This is quite straightforward for blanket materials, where the dopant and matrix are usually chemically different. However, when dealing with more complex samples like the ones reported in Figure 1a–d, such a conventional approach is no longer applicable. In fact, such a patterned sample displays B-doped Si fins surrounded by B-doped SiO_2_; therefore, a SIMS experiment would return secondary ions simultaneously for both areas, hampering the discrimination of the dopant implant on the region of interest, in this study the silicon fins. Therefore, in this case, alternative methods are needed for the correct quantification of the boron dopant in the silicon fins.

### 3.1 1.5D SIMS Approach

One option to obtain information on one specific area is to selectively etch away the areas and materials in which one is not interested and proceed with the analysis of the material of interest (1.5D SIMS approach).^15^

Since the purpose of this study is the quantification of the boron implanted in the silicon fins, an etching selective to the SiO_2_ was performed. In this way, any contribution from the SiO_2_ is removed, and the sample can be regarded as boron-implanted silicon. However, knowledge of the geometry of the structures and the related coverage is needed to account for the detection of the matrix signal (i.e., ^30^Si^+^) from the fins and the bulk simultaneously. In this specific case, cross-sectional TEM was performed to obtain information about the geometry of the silicon fins and their surface coverage, which was found to be ∼10% of the total surface for all fin widths.

Using B^+^ as the dopant signal and ^30^Si^+^ as the matrix signal, the boron implant profile was obtained (see Figure 2a), and it showed good agreement with the SRIM simulation displayed in Figure 1f for silicon. However, a dip is observed where the top of the implant is expected, as one can notice from Figure 2a. This is most likely related to some damage occurring during the implantation, usually very pronounced where the top of the implant is supposed to be,^25^ which can even worsen during laser annealing, as discussed in the following sections. However, if the implant curve is interpolated between the unaffected regions (see dashed line in Figure 2a), a peak concentration just below 9 × 10^20^ at/cm^3^ is found, again in good agreement with the SRIM simulation.

**Figure 2:**
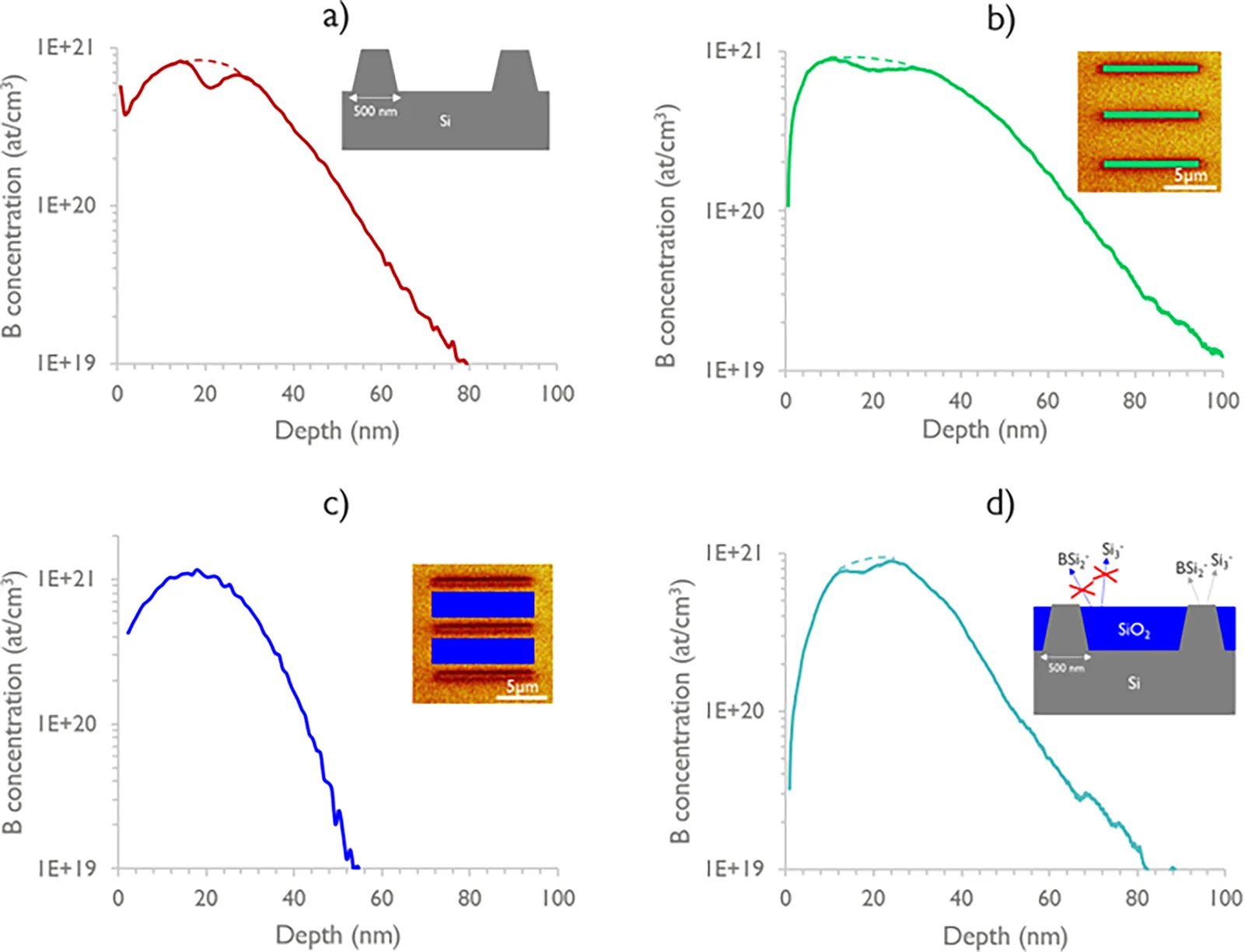
Quantified SIMS depth profiles (boron in silicon) for the 500 nm wide fin in the laser annealed sample obtained with the 1.5D SIMS (a), high lateral resolution SIMS (b), and SF-SIMS (d) approaches. Boron quantification for the silicon oxide obtained with the high lateral resolution SIMS approach (c).

Although the 1.5D SIMS approach leads to a correct boron quantification, in line with SRIM, it requires meticulous sample preparation for the selective removal of the oxide, together with supporting characterization techniques to collect accurate knowledge of the geometry of the structures, such as TEM analysis. Moreover, correction factors must be derived to correctly interpret the obtained results, and unprecise surface coverage will be directly reflected in erroneous concentration. Additionally, for smaller fin dimensions, the etching process might not be completely effective and lead to collapse of the fins, which will again lead to incorrect quantification due to underestimated or overestimated signal intensities of both boron and matrix signals. It should also be noted that the applicability of the proposed 1.5D SIMS approach to more complex heterostructures, such as III–V semiconductor systems, may be limited by the availability of sufficiently selective chemical etching procedures capable of preserving the structures of interest prior to SIMS analysis.

### 3.2 High Lateral Resolution SIMS Approach

An alternative approach to quantify the boron in the silicon fins only, foresees the use of a focused analysis beam which enables high lateral resolution (i.e., beam spot size of <200 nm) and the possibility to separate the fins region from the oxide region. For this kind of approach, no sample preparation is required; however, the areas of interest must be within the analytical beam spot size. In fact, as shown in the inset in Figure 2b, the analysis is performed on an area larger than the area where the fins are confined (15 × 15 μm^2^) containing both silicon fins and silicon oxide; then, regions of interest (ROIs) are selected on the silicon fins only (green regions). In this way, boron quantification on only the silicon fins is enabled, leading to a peak concentration of ∼9 × 10^20^ at/cm^3^ (see Figure 2b), again in good agreement with SRIM simulations. Moreover, taking advantage of the high lateral resolution achieved in this operational mode, it was possible to verify that indeed boron was implanted also in the surrounding SiO_2_, as expected. Its quantification is reported in Figure 2c (ROIs defined in the SiO_2_ region shown in blue) and is again in good agreement with the SRIM simulation displayed in Figure 1f for silicon oxide. Unfortunately, this approach is not applicable to fin widths below 100 nm, due to lateral resolution limitations.^26^ In practice, even when the lateral resolution is sufficient, larger structures (i.e., >100 nm) may still be affected by lateral resolution limitations. In particular, accurate positioning of the ROIs on the fin regions can be challenging, and sensitivity may be reduced due to the limited number of available pixels.

It is noteworthy that, also for the quantification with the high lateral resolution approach of the largest fins, i.e., 500 nm, a dip at the top of the implant is detected and therefore not related to the SIMS quantification methodology used.

### 3.3 SF-SIMS Approach

The two approaches described above allowed, to some extent, the quantification of the boron dopant in the 500 nm wide silicon fins. However, a more general and effective approach is needed in order to avoid long sample preparation and extend the quantification to smaller fins. In this context, the self-focusing SIMS (SF-SIMS) concept has been demonstrated as a valuable approach for quantification in confined volumes and nanometric dimensions.^10–13^ The principle of SF-SIMS relies on the fact that a cluster ion can only be emitted if its constituent atoms are already in close proximity in the point of origin.^14^ In this way, the information is “self-focused” to the region of interest. In this study, the SF-SIMS concept foresees the formation of two clusters that are specific for the boron (dopant) and the silicon (matrix). The paramount condition for these two clusters to be used as self-focused ions is that they are only formed in the silicon fins and not detected in the oxide region, as schematically depicted in Figure 3a.

**Figure 3:**
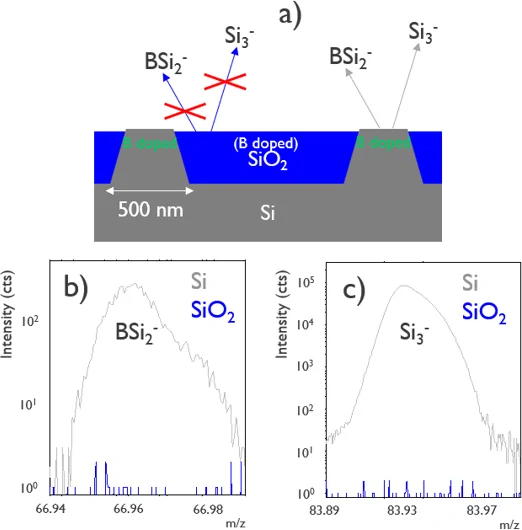
(a) Schematic description of the SF-SIMS concept; dopant (b) and matrix (c) cluster ions are detected exclusively in the silicon reference (gray lines).

In order to find the suitable clusters, the mass spectra of a silicon and a silicon oxide standard implanted with the same boron dose were compared and screened. It was assessed that among the possible B_*x*_Si_*y*_ clusters, characteristic for the B-implanted silicon fins, the cluster BSi_2_^–^ was detected only in the silicon fins and not in the oxide (see Figure 3b). Regarding the cluster related to the matrix, Si_3_^–^ was chosen as the best compromise between intensity and saturation, for its consistency with the B-containing cluster (same number of atoms), and its detection only in the silicon fins (see Figure 3c).

Using the above-mentioned self-focused clusters, it was possible to carry out boron quantification not only on the 500 nm wide fins (Figure 2d), for which a very good agreement with the other two approaches was found, but also on all fin dimensions down to a 20 nm width (Figure 4a).

**Figure 4:**
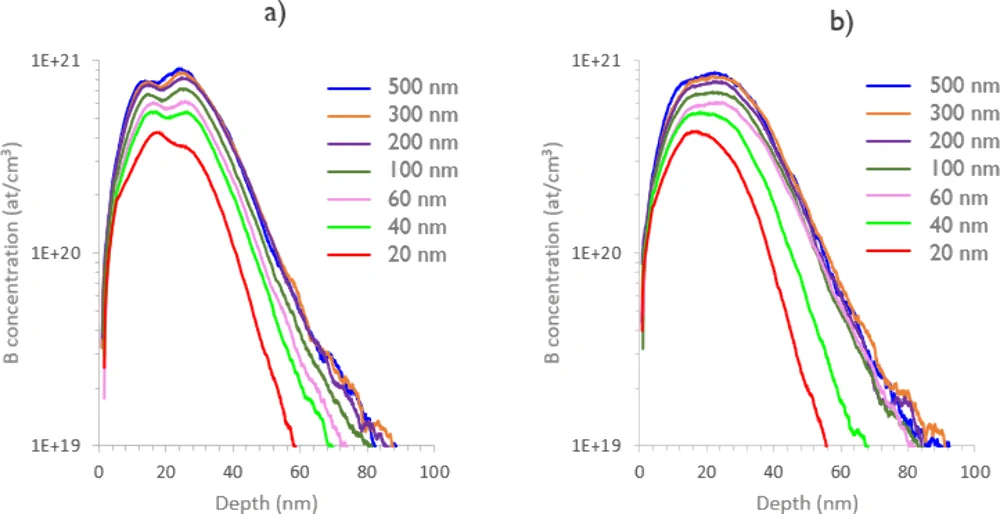
Boron quantification obtained using the self-focused cluster ion BSi_2_^–^. (a) Quantified SIMS depth profiles overlay for different fins widths (from 500 to 20 nm) of the laser annealed sample. (b) Quantified SIMS depth profiles overlay for different fins widths (from 500 to 20 nm) of the non-annealed sample.

Interestingly, the boron concentration does not remain constant, as one would expect from to the implant process; instead, it decreases with the fin width, going from a peak concentration of ∼9 × 10^20^ at/cm^3^ for the 500 nm wide fins to ∼4.5 × 10^20^ at/cm^3^ for the 20 nm wide ones. This trend was observed in the non-annealed fins as well, as shown in Figure 4b. Therefore, the annealing step cannot be considered the cause of such a trend.

This behavior might be related to two possible situations. On the one hand, some boron out-diffusion from the fins to the oxide might occur. In this case, the amount of boron in the silicon fins would decrease, diffusing into the oxide, and therefore, a lower concentration of boron would be detected in the fins. On the other hand, we should consider that the silicon fins exhibit sidewalls (points of contact with the surrounding oxide), which can be regarded as Si/SiO_2_ interfaces. In the sidewall region, the cluster formation would hence be affected by the presence of oxygen, reducing (or even suppressing) the formation probability of BSi_2_^–^ and Si_3_^–^ self-focused clusters. This would result in a lower intensity for the two clusters of interest. In any case, the effect would be more pronounced for smaller fins because the boron diffusion length would be apparently larger for smaller widths and/or because the number of sidewalls would also be greater in the SIMS measurement field-of-view compared to larger fins.

An analogous trend in concentration with fin dimensions was somehow observed by Folkersma et al.^27^ on a similar sample via micro four-point probe (μ4PP) analysis. In this study, the resistance of the fins measured as a function of the fin dimensions increased with decreasing fin width. It is important to mention that μ4PP, conversely to SIMS, does not measure the total dopant concentration; it instead measures the active carrier. In this study it was demonstrated that the trend described by μ4PP experiments was related to the presence of amorphous areas around the core of the fins, which are generated after boron implantation and do not recover even after annealing. In the amorphous regions, indeed, scanning spreading resistance microscopy (SSRM) showed a resistance ∼4 orders of magnitude higher compared to the one measured in the core of the fins. As this effect is closely related to implant-induced defects at the sidewalls, it suggests that the fin sidewalls may play an important role in SF-SIMS quantification.

As shown in Figure 2a,b,d, the three different SIMS approaches for boron quantification in the fins led to depth profiles characterized by a dip at the top of the implant. Dark-field scanning transmission electron microscopy (DF-STEM) cross-sectional imaging of the laser annealed 500 nm wide fins (Figure 5a) reveals a markedly brighter region located ∼20 nm below the surface compared to the rest of the fin, suggesting the presence of a highly defective zone. SIMS depth profiles obtained on the same fins in high lateral resolution and negative polarity mode indicate the presence of oxygen within this defective region identified by DF-STEM (Figure 5b).

**Figure 5:**
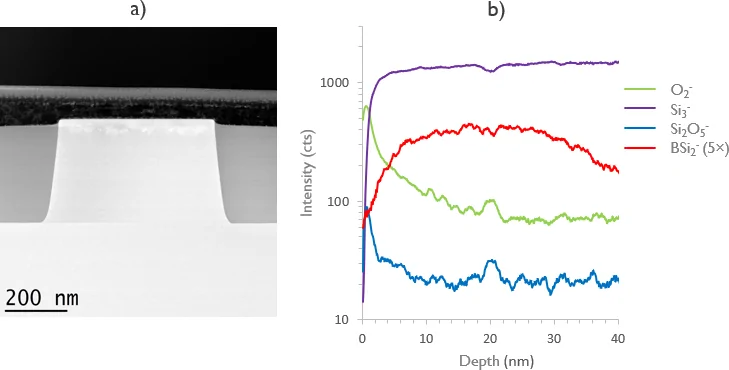
(a) DF-STEM cross section on the laser annealed 500 nm wide fins. (b) Negative polarity intensity depth profile (high lateral resolution mode) recorded on the silicon fins in the same sample as (a) to follow the oxygen and silicon trends in the bright region identified in (a). The cluster ion used in this work for the boron quantification (BSi_2_^–^) is also reported with scaled intensity, multiplied 5×.

In the profile it is possible to observe that, at ∼20 nm where the top of the implant is expected, oxygen-containing ions show higher intensity, while the self-focused ions used in this study for the quantification show the opposite trend. In this region, therefore, the intensity collected for the cluster ions of interest is underestimated since we proved that the two cluster ions used for the SF-SIMS approach, i.e., BSi_2_^–^ and Si_3_^–^, would form only in a silicon matrix and were not detected in a SiO_2_ matrix. These observations explain the discrepancy between the SF-SIMS quantification and the expected theoretical behavior at the top of the implant. Oxygen is most likely incorporated within these defective regions, likely diffusing from the surrounding oxide into the fins, thereby affecting the accuracy of the SF-SIMS quantification.

However, the presence of oxygen also explains the dip observed in the quantified depth profiles obtained with the other two more traditional approaches used for the 500 nm wide fins (Figure 2a,b). In fact, the ionization yield of an ion is strongly dependent on its matrix.^28–32^ The presence of oxygen at ∼20 nm from the surface is indeed responsible for a change in the matrix, and therefore, the intensity of the signals used for the quantification will be affected accordingly, leading to an “incorrect” quantification in the concerned region even when non-self-focused ions are considered. The presence of the dip was not observed in non-annealed fins, as reported in Figure 4b for all the fin widths available. This strongly supports the theory that the post laser anneal might be responsible for this kind of defect in the fins.

This study demonstrates that the SF-SIMS approach provides an efficient and relatively fast means of dopant quantification in structures with critical dimensions below 500 nm, with a measurement time not exceeding 40 min per analysis under the experimental conditions employed in this work and without requiring specific sample preparation or specialized SIMS instrumentation. While some questions remain regarding the mechanisms underlying the observed concentration trends with decreasing fin dimensions, this approach presently appears to be the only viable method for dopant quantification in state-of-the-art device architectures. Complementary techniques, such as atom probe tomography, could help elucidate the origin and extent of the SF-SIMS limitations and support the development of correction or normalization protocols for SF-SIMS data.

## 4 Conclusion

This study established SF-SIMS as a successful approach for the quantification of dopants in structures featuring lateral dimensions on the order of tenths of nanometers. Specifically, SF-SIMS was applied to a structured boron-doped device, composed of silicon fins (with width size ranging from 500 to 20 nm) surrounded by silicon oxide. As both areas, i.e., silicon fins and surrounding silicon oxide, were doped with boron due to the implantation process used, it was necessary to find an approach that could clearly discriminate between the two areas. More traditional approaches, such as 1.5D SIMS and high lateral resolution SIMS, were demonstrated to be effective but not direct or universal. On the contrary, with the SF-SIMS approach, the spatial resolution limitation of conventional SIMS was overcome without sacrificing sensitivity. This was achieved by selecting specific cluster ions that originated only from the region of interest, i.e., the silicon fin region. Our approach pointed out the presence of a boron implant with a peak concentration of ∼9 × 10^20^ at/cm^3^ for the widest silicon fins (i.e., 500 nm), in good agreement with SRIM simulations and the two other SIMS approaches mentioned earlier. However, for smaller fins, the boron concentration was found to be lower, with the lowest value recorded for the 20 nm wide fins. We postulated some rational explanations for this discrepancy in boron concentration, contemplating either boron diffusion toward the surrounding oxide and/or effects due to the SiO_2_/Si sidewalls. Although supplementary studies are needed to clarify this difference in boron concentration, this study demonstrates that SF-SIMS can be already regarded as an efficient approach to quantify dopants in very confined volumes with high sensitivity and without requiring specific sample preparation.
